# Modified thoracoscopic wedge resection of limited peripheral lesions in S10 for children with congenital pulmonary airway malformation: Initial single-center experience

**DOI:** 10.3389/fped.2022.934827

**Published:** 2022-08-18

**Authors:** Rui Guo, Yunpeng Zhai, Shisong Zhang, Huashan Zhao, Hongxiu Xu, Longfei Lv

**Affiliations:** ^1^Department of Thoracic and Tumor Surgery, Children’s Hospital Affiliated to Shandong University, Jinan, China; ^2^Department of Thoracic and Tumor Surgery, Jinan Children’s Hospital, Jinan, China

**Keywords:** children, congenital pulmonary airway malformation, pneumonectomy, posterior basal segment, thoracoscopy

## Abstract

**Objective:**

The present study aimed to evaluate the safety and feasibility of modified thoracoscopic wedge resection of limited peripheral lesions in the posterior basal segment (S10) in children with congenital pulmonary airway malformation (CPAM).

**Materials and methods:**

We retrospectively analyzed the clinical data of children with CPAM who underwent thoracoscopic modified wedge resection at our institution from November 2020 to February 2022. The surgical method was as follows: we marked the external boundary of the lesion with an electric hook, dissected and retained the segmental vein between the lesion and normal lung tissue as the internal boundary, cut the arteries, veins, and bronchus entering the lesion, and cut and sealed the lung tissue between the internal and external boundaries with LigaSure™ to complete the modified wedge resection.

**Results:**

A total of 16 patients were included, aged 3.8−70.0 months and weighing 6.5−21.0 kg. The intraoperative course was uneventful in all patients. The median operation time and intraoperative bleeding volume were 74 min (50−110 min) and 5 mL (5−15 mL), respectively. The median postoperative drainage tube indwelling time was 3 days (2−4 days), and the median postoperative hospital stay was 6 days (4−8 days). Pathological diagnosis included two cases of type 1, 10 cases of type 2, and four cases of type 3 CPAM. There were no cases of intraoperative conversion, surgical mortality, or major complications. However, subcutaneous emphysema occurred in two children, which spontaneously resolved without pneumothorax orbronchopleural fistula development. All patients were followed up for a median period of 10 months (3–18 months), and there were no cases of hemoptysis or residual lesions on chest computed tomography.

**Conclusion:**

Modified thoracoscopic wedge resection *via* the inferior pulmonary ligament approach is safe and feasible for children with CPAM with limited peripheral lesions in S10.

## Introduction

Congenital pulmonary airway malformation (CPAM) is a rare hamartoma composed of abnormal lung tissue and varying degree of cystic changes. It is characterized by abnormal airway pattern during the formation of the bronchial branches and formed by abnormal branches of immature bronchioles (1). At present, most CPAM cases can be diagnosed before delivery. Due to the risk of recurrent respiratory tract infections and carcinoma, postpartum surgical resection is the preferred treatment, and the overall prognosis is good (2, 3).

In recent years, with the improvement of minimally invasive technology, thoracoscopic treatment of CPAM has gradually become the mainstream. The common surgical methods include lobectomy, wedge resection, and segmental resection (4, 5). Although thoracoscopic lobectomy is a recognized treatment method, lung-sparing surgery such as analytical segmentectomy or wedge resection may be suitable if the lesion is small and localized, and there are no inflammatory changes. Thoracoscopic resection of simple lung segments is similar to thoracoscopic lobectomy, and the operation is relatively easy; however, thoracoscopic resection of complex lung segments, particularly the posterior basal segment (S10), is extremely challenging. For lesions with such complex locations, thoracoscopic wedge resection is the most commonly selected method (6, 7).

For limited peripheral lesions in S10, thoracoscopic wedge resection is a simple and effective surgical method, but there is a risk of residual lesions and persistent air leaks (3). The reason is that CPAM is a cystic lesion with non-linear internal boundary, while wedge resection involves linear cutting. If the resection edge is insufficient, the lesion will remain. Additionally, if the resection edge is too deep, the incisional margin becomes wider, thereby increasing the risk of persistent air leaks. By analyzing the anatomical characteristics of CPAM, we found that these lesions occur within the lung segment, and the intersegmental vein is the natural boundary of the lung segment (8). We hypothesized that taking the segmental vein adjacent to the lesion as the internal boundary for cutting can minimize the occurrence of postoperative hemoptysis and lesion residue. To the best of our knowledge, there is no relevant research on this topic.

Therefore, we modified the thoracoscopic wedge resection procedure by taking the adjacent segmental vein as the internal boundary, and in this study, we evaluated the feasibility and effectiveness of this surgical method in children with CPAM with limited peripheral lesions in S10.

## Materials and methods

### Study design and patients

The present study adhered to the tenets of the Declaration of Helsinki and was approved by the Ethics Committee of our hospital (the ethical approval number: SDFE-IRB/T-2022047). Additionally, written informed consent was obtained from the parents of the patients.

This study was a retrospective review of the clinical data of children with CPAM who underwent modified thoracoscopic wedge resection at our institution from November 2020 to February 2022. The inclusion criteria were as follows: (1) diagnosis of CPAM; (2) limited peripheral lesions located in unilateral S10, without any episode of infection; (3) maximum lesion diameter of ≥ 2 cm; and (4) no prior surgical treatment. The exclusion criteria were the following: (1) concomitant diseases, such as congenital heart disease; (2) immunocompromised state; (3) restrictive or obstructive chest wall disease; and (4) multiple lesions.

Preoperative enhanced computed tomography (CT) was used to confirm the diagnosis, identify the lesions, and determine the route of the intersegmental veins. Routine examinations included standard electrocardiography, echocardiography, and blood tests. Patient demographics, CPAM characteristics (location and pathological type), and perioperative data (operation time, blood loss, drainage tube indwelling time, complications, and postoperative hospital stay length) were recorded for all patients.

### Surgical technique

**FIGURE 1:**
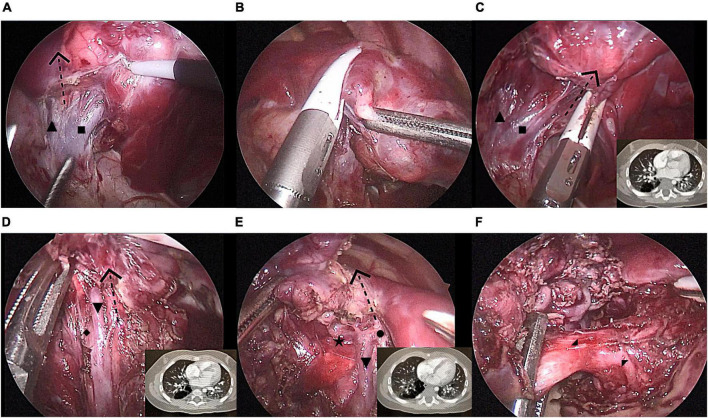
Modified thoracoscopic wedge resection of limited peripheral lesions in the right S10. **(A)** Separating the inferior pulmonary vein and its branches [V6, common basal vein (CBV)] and determining the left boundary of the lesion (V6); **(B)** Cutting and closing the left lesion boundary with LigaSure™ along V6; **(C)** Cutting and closing the lower part of the right lesion boundary with LigaSure™ along CBV; **(D)** Cutting and closing the middle part of the right lesion boundary with LigaSure™ along V9 and V10; **(E)** Cutting and closing the upper part of the right lesion boundary with LigaSure™ along V9; **(F)** Separation of the diseased bronchus and artery (B10 and A10) entering the lesion. CBV, common basal vein: ■, V^6^:▲, V^9+10^:▼, V^8^:◆, V^9^:•, V^10*b*^:★, B^10^:◢, A^10^:◤.

The patients were positioned in the full lateral decubitus position, and the surgeon was always at the ventral side of the patient. One-lung ventilation was achieved under single lumen endotracheal intubation (the bronchial occluder selectively blocks the main bronchus on the affected side). Due to the narrow chest space of children, it is difficult to obtain the ideal operation space only by one-lung ventilation. Therefore, after the completion of one-lung ventilation, the chest is initially insufflated with a low flow rate and low pressure of CO_2_ to help complete the lung collapse. A flow rate of 1–2 L/min and pressure of 4–8 mmHg are maintained throughout the cases. The observation port was located at the midaxillary line in the 8th intercostal space, and the two surgical ports were located at the anterior axillary line in the 8th intercostal space and the subscapular line in the 7th intercostal space. A rigid 30-degree 5-mm optic thoracoscope was used for vision (Figure 3). The affected lung was gently pulled and squeezed with the operating forceps to collapse it as much as possible, and the electric hook was used to mark the external boundary of the lesion. The lower pulmonary ligament was cut, the lower pulmonary vein exposed, and the lower pulmonary vein and its branches [V6, common basal vein (CBV)] fully freed using the electric hook. The lung tissue between V6 and the external boundary of its corresponding lesion was cut and closed with a 5-mm/37-cm Ligasure™ (Maryland Jaw Laparoscopic Sealer/Divider; Covidien LLC., Mansfield, MA, United States) along the near S10 edge of V6 (Figures 1A,B). The CBV and its branches were dissected according to the direction of the vein branches on enhanced chest CT. The intra-segmental vein entering the lesion was cut, and the intra-segmental vein between the lesion and normal lung tissue was retained as the internal boundary. The lung tissue between the internal boundary (segmental vein) and the corresponding external boundary of the lesion was cut and closed with Ligasure™ (Figures 1C–E). Next, the bronchus and artery entering the lesion were located, separated, ligated, and cut (Figure 1F). The diseased lung tissue was removed using the extraction bag and the pleural cavity was washed with warm normal saline. The cut edges of the blood vessels, bronchus, and lung tissue were carefully examined, and after confirming that there was no active bleeding or air leakage, they were closed with a 5-0 Prolene suture. After the lungs were re-expanded, a closed thoracic drainage tube was placed through the surgical port at the anterior axillary line in the 8th intercostal space. The chest tube was removed when there was no air leak, and the amount of daily drainage was <20 ml.

**FIGURE 2:**
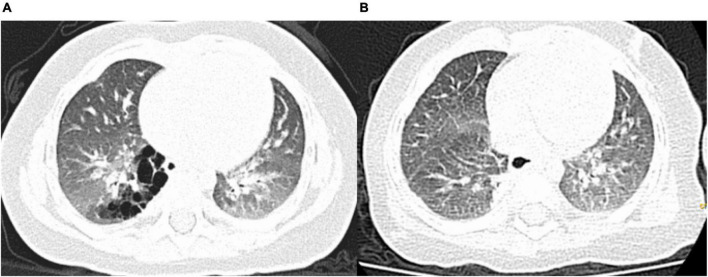
Pre- and postoperative computed tomography (CT) findings. **(A)** Preoperative CT showing a congenital pulmonary airway malformation in the right S10; **(B)** Follow-up CT scan obtained 3 months after the operation showing no residual lesions.

**FIGURE 3:**
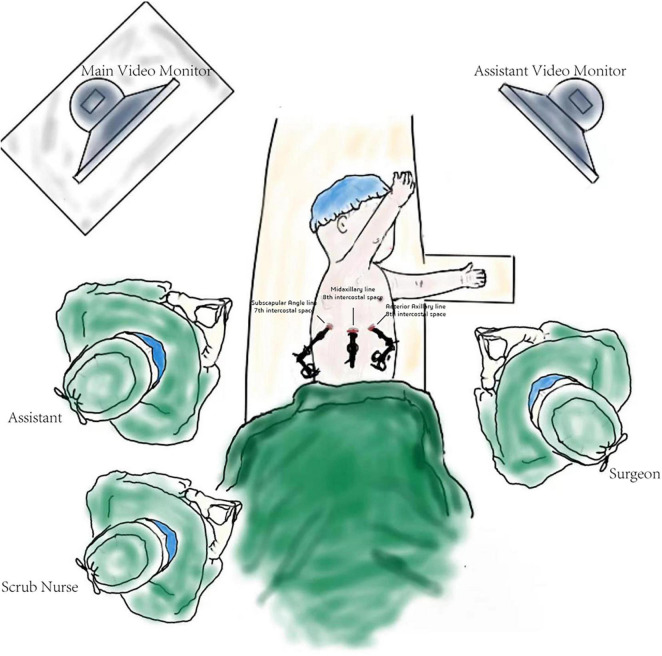
Schematic diagram of the operation.

### Statistical analysis

Continuous data were presented as medians and interquartile ranges, while categorical data were presented as frequencies and percentages. IBM SPSS Statistics version 23.0 (IBM Co., Armonk, NY, United States) was used for all statistical analyses.

## Results

### Medical history and diagnosis

Clinical parameters are shown in Table 1. After excluding two children with obvious pulmonary infection (each lung inflammation leads to unclear lesion boundary), 16 children (12 males and 4 females) with a median age of 7.2 months (range, 3.8–70.0 months) and a median body weight of 9.0 kg (6.5–21.0 kg) were included in the analysis. Among them, 15 were diagnosed with CPAM by prenatal color Doppler ultrasound and the diagnosis was confirmed by chest CT within 1 month after birth. One child was diagnosed by chest CT due to recurrent respiratory tract infections. The lesions were located in the right S10 in 12 cases and the left S10 in four cases. The maximum lesion diameter was 3.2 cm (2.3–4.5 cm). Seven children had respiratory symptoms (five cases of cough and two cases of asthma), and nine children were asymptomatic.

**TABLE 1:** Clinical data of children undergoing modified thoracoscopic wedge resection.

Patient number	Gender	Age (months)	Weight (kg)	Systematic (Y/N)	Location (L/R)	Operative time (min)	Blood loss (ml)	Drainage duration (days)	Duration of post-operative hospital stay (days)	Pathological type	Complications	Follow-up time (months)
1	M	6.3	9.0	N	R	90	10	2	4	2	N	3
2	F	5.9	7.0	N	R	85	10	3	6	2	N	3
3	M	4.3	7.0	Y	R	65	5	3	5	3	N	3
4	F	3.8	8.0	N	R	69	5	3	6	3	N	6
5	M	4.6	6.5	N	L	60	5	4	8	2	N	6
6	M	8.5	9.5	Y	L	73	5	3	6	2	N	8
7	M	5.4	9.0	N	R	70	5	3	6	2	N	9
8	M	10.4	11.8	Y	R	80	5	4	7	1	N	10
9	F	7.2	9.0	Y	R	78	10	3	6	2	N	10
10	M	7.4	9.0	N	R	60	5	4	6	3	N	10
11	F	20.0	14.0	N	R	50	5	3	5	2	N	11
12	M	3.8	8.5	Y	R	103	5	3	6	2	N	12
13	M	10.2	10.0	N	R	75	10	3	5	2	N	13
14	M	7.2	10.5	N	L	62	5	4	7	2	Subcutaneous emphysema	14
15	M	70.0	21.0	Y	R	110	10	3	6	1	N	16
16	M	31.0	16.5	Y	L	100	15	4	7	3	Subcutaneous emphysema	18

### Intraoperative conditions and postoperative management

The intraoperative course was uneventful for all 16 patients. The median operation time and intraoperative bleeding volume were 74 min (50–110 min) and 5 mL (5–15 mL), respectively. The median postoperative drainage tube indwelling time was 3 days (2–4 days), and the median postoperative hospital stay was 6 days (4–8 days). Pathological diagnosis included two cases of type 1, 10 cases of type 2, and four cases of type 3 CPAM. There were no cases of intraoperative conversion, surgical mortality, or major complications. However, subcutaneous emphysema occurred in two children, which spontaneously resolved without pneumothorax or bronchopleural fistula development (Table 1).

### Follow-up

Follow-up examinations and chest CT were performed at 3, 6, and 12 months after surgery. All patients were followed up for a median period of 10 months (3–18 months), and there were no cases of hemoptysis or residual lesions on chest CT (Figures 2A,B).

## Discussion

In this preliminary study, we selected 16 children with CPAM diagnosed by enhanced chest CT with limited peripheral lesions located in a unilateral S10 to verify the feasibility and effectiveness of modified wedge resection under thoracoscopy, and achieved good clinical results.

Congenital pulmonary airway malformation, formerly known as congenital cystic adenomatoid malformation, is the most common congenital cystic degeneration of the lungs (9). With the development of minimally invasive technology, thoracoscopic lobectomy has become the main surgical method for the treatment of CPAM. For localized peripheral lesions, although lobectomy can completely remove the lesions, it leads to the loss of a large amount of normal lung tissue, which is not conducive to the recovery of lung function and long-term prognosis (6, 10). Some experts have proposed lung-sparing surgery, which aims to maximize the preservation of normal lung tissue after resecting lesions. At present, lung-sparing surgical methods include analytical segmentectomy and wedge resection (9, 11). Analytical segmentectomy is an ideal surgical method. Simple analytical segmentectomy refers to the resection of the lingula, basal, dorsal, or left upper lobe proper segments. The anatomical structure of these segments is similar to pulmonary fissure fusion, and it is easier to remove lung segments under thoracoscopy. Complex analytical segmentectomy, defined as the resection of lung segments involving more than two intersegmental planes, is more difficult to perform under thoracoscopy, in particular, S10 resection (12, 13). S10 is located distant from the hilum, the segmental hilum is located in the deep part of the lung tissue, and the shape of the plane between the segments is irregular (14). In children with CPAM, the segmental bronchus is small, the Koch foramen is immature, and the segmental or subsegmental bronchi often have atresia, making it more difficult to accurately determine the intersegmental plane (8, 15). Therefore, thoracoscopic S10 resection is challenging and not applicable to all children with CPAM. At present, thoracoscopic wedge resection is still the main surgical method for limited peripheral lesions in S10.

Although thoracoscopic wedge resection has achieved satisfactory clinical results in the treatment of CPAM in children, complications such as lesion residue and persistent air leaks are still inevitable (3, 7). CPAM is a cystic lesion with irregular edges convex to the inside, while wedge resection involves linear resection. If the resection edge is insufficient, it will cause residual lesions. Additionally, if the resection edge is too deep, the incisional margin becomes wider, thereby increasing the risk of persistent air leaks. As CPAM is a benign lesion, it is only required to completely resect the lesion, and it is not necessary to consider the safe distance of the resection edge like in patients with lung cancer. Because the external boundary of peripheral lesions is very obvious, determination of the internal boundary is extremely important (9). CPAM is a hamartoma-like lesion caused by excessive hyperplasia at the level of bronchioles and terminal bronchi and impaired development of pulmonary acini. Since the lesion is located within the lung segment, it is feasible to take the intersegmental vein as the internal boundary (8). Therefore, it is feasible that resection along the segmental vein surrounding the lesion can not only ensure the integrity of the lesion resection, but also preserve the normal lung tissue to the greatest extent. This surgical method is modified on the basis of the original thoracoscopic wedge resection, and pays more attention to the determination of the internal boundary of the lesion. Therefore, we named this surgical method “modified thoracoscopic wedge resection.” Compared with “common” wedge resection, our surgical method uses the intersegmental vein adjacent to the lesion as the internal boundary for cutting instead of linear resection. As a result, the risk of residual lesions, which may be caused by insufficient cutting, cutting more normal lung tissue and intersegmental vein injury by deep cut-off, are minimized. Stem-branch technique considers the bronchus as the key structure. In this method, we should find the stem of the basic segmental bronchus on the back of the inferior pulmonary vein, dissociated the branches of the basic segmental bronchus and determined the target segment bronchus to complete the determination of the intersegmental plane. This method has achieved good results in segmentectomy, but is not applicable to the resection of the limited peripheral lesions in S10. Because it will not only lose too much normal lung tissue, but also may lead to bleeding or air leakage for the extensive dissociation. Our surgical method considers the segmental vein adjacent to the lesion as the key structure. We cut the lesion when the intersegmental vein is taken as the internal boundary and then the normal lung tissue can be preserved to the greatest extent. This method is more suitable for limited peripheral lesions in S10.

Unlike arteries and bronchi, pulmonary veins exhibit many variations. Accurately determining the segmental veins adjacent to the lesion and outlining the internal boundary of the lesion is the key to the success of the modified thoracoscopic wedge resection. We considered the following two aspects. First, with the improvement in imaging technology and diagnostic level, the segmental vein can be clearly displayed. The operator can clarify the venous variation and its position relationship with the lesion through preoperative film reading, outline the boundary of the lesion through the segmental vein, and design the resection path of the internal edge of the lesion (16, 17). Second, according to the traditional hilar approach, the vein is located in the deep surface of the artery and bronchus, which makes it difficult to expose it for dissection. In addition, the vein is a reflux vessel located deep in the lung tissue and close to the hilum. Therefore, it is very difficult to retrogradely track the main vein through the terminal vein. Exposure of the vein and its branches through the inferior pulmonary ligament approach is relatively simple: the inferior pulmonary vein can be clearly exposed after cutting the inferior pulmonary ligament, and the inferior pulmonary vein branches can be easily separated by blunt separation along the adventitia (18, 19).

Modified thoracoscopic wedge resection can minimize the risk of postoperative hemoptysis and residual lesions. Our patients were followed up for 3–18 months. There were no cases of hemoptysis or residual lesions on CT, and a good short-term follow-up effect was achieved. Since this method is not a strict segmental resection, there is a risk of cross-sectional air leakage (4, 11). We have taken the following measures. First, after the operation, warm normal saline was injected to soak the lung section into water and the anesthesiologist was asked to drum the lung. If there is air leakage, the exact position should be determined and sutured with 5-0 Prolene. Second, closure was performed in all children with a 5-0 Prolene suture. Through the above efforts, among the 16 children who underwent modified thoracoscopic wedge resection, only two developed subcutaneous emphysema, which spontaneously resolved without pneumothorax or bronchopleural fistula development.

The smooth progress of this operation depends on the accurate dissection of the adjacent segmental vein. If an inflammatory process exists in the affected lung tissue, it may be difficult to dissect and expose the interstitial vein in the middle of the operation. Hence, children with inflammatory lung processes are not suitable for modified wedge resection under thoracoscopy. In children without symptoms before operation and no inflammatory reaction in the lungs, thoracoscopic modified wedge resection can be performed smoothly. However, we should pay attention to the screening of children with respiratory symptoms before operation. We adopted the following steps. The first step was to carefully read the preoperative CT scans and exclude children with a severe inflammatory reaction involving the lung tissue and unclear lesion boundaries. The second step was that the children screened in the first step were observed by thoracoscopy during the operation. If there is a chronic inflammatory reaction in the affected lung tissue, which would cause adhesions near the intersegmental vein and difficulty in separating it, the operation method should be changed in time and thoracoscopic lobectomy should be adopted. In such cases, performing the modified thoracoscopic wedge resection is more likely to result in residual lesions, pneumothorax, and bronchopleural fistulas. In this study, the operation was successfully completed in nine asymptomatic children. Two children were excluded because the preoperative CT scans showed severe pulmonary inflammation and unclear lesion edges. In the remaining seven children, there were no clinical symptoms of wedge-shaped venous lesions involving the lower respiratory tract before thoracoscopy, and there was no severe inflammation involving the middle and lower respiratory tract. The reason that there were no cases of surgical method change in our study may be due to the fact that the patients were relatively young, mostly infants, with a low possibility of chronic lung inflammation.

Our center successfully completed modified thoracoscopic wedge resection in 16 cases with good short-term clinical results. However, this study also had some limitations. It was a single-center retrospective study, and the sample size was small. Thus, it is necessary to verify the advantages of this surgical method in future prospective and multicenter studies with larger samples. The key point of this operation method is to determine the internal boundary of the lesion (segmental vein). It is not easy to have a clear understanding of the route of the vein and its relative position with the lesion by preoperative CT, which depends on the operator’s CT reading ability, or to accurately dissect the corresponding vein during the operation (20). Based on the original image data, three-dimensional reconstruction can reconstruct the plane image data in three-dimensional form through software to more clearly present the original shape and position relationship of tissues and organs, avoiding a possible error caused by the process of brain imagination reconstruction. Three-dimensional reconstruction has been widely used in adult lung diseases, but it is difficult to perform in children because their pulmonary vessels and bronchi are thin (21). In the future, it is expected to be further improved with the upgrading of the production software. Three-dimensional reconstruction is also very helpful to judge the positional relationship between the lesion and the adjacent vein. When dissecting and looking for specific blood vessels and bronchi during operation, the three-dimensional image can be “reduced” into a “linear structure” to simplify the anatomical presentation of blood vessels and bronchi and aid in accurately finding the relevant blood vessels and bronchus for processing.

In conclusion, the modified thoracoscopic wedge resection via the inferior pulmonary ligament approach is safe and feasible for children with CPAM with limited peripheral lesions in S10.

## Data availability statement

The original contributions presented in the study are included in the article/supplementary material, further inquiries can be directed to the corresponding author.

## Ethics statement

The studies involving human participants were reviewed and approved by Committee of Children’s Hospital Affiliated to Shandong University and Jinan Children’s Hospital. Written informed consent to participate in this study was provided by the participants’ legal guardian/next of kin. Written informed consent was obtained from the individual(s), and minor(s)’ legal guardian/next of kin, for the publication of any potentially identifiable images or data included in this article.

## Author contributions

RG participated in clinical practice, contributed to the collection and analysis of data, and grafted and revised the manuscript. YZ carried out data collection. SZ carried out patient recruitment and clinical practice, contributed to the conception and design of the study, and drafted and revised the manuscript. HZ and HX participated in clinical practice. LL helped in the study design and drafting of the manuscript. All authors read and approved the final manuscript.

## Abbreviations

CBV: common basal vein
CPAM: congenital pulmonary airway malformation
CT: computed tomography
S10: posterior basal segment.
